# Source reconstruction of airborne toxics based on acute health effects information

**DOI:** 10.1038/s41598-018-23767-8

**Published:** 2018-04-04

**Authors:** Christos D. Argyropoulos, Samar Elkhalifa, Eleni Fthenou, George C. Efthimiou, Spyros Andronopoulos, Alexandros Venetsanos, Ivan V. Kovalets, Konstantinos E. Kakosimos

**Affiliations:** 1 Department of Chemical Engineering and Mary Kay O’Connor Process Safety Center, Texas A&M University at Qatar, Education City, Doha, PO Box 23874, Qatar; 2 Qatar Biobank for Medical Research, Doha, 5825 Qatar; 3 Environmental Research Laboratory, INRASTES, NCSR Demokritos, Patriarchou Grigoriou & Neapoleos Str, 15310 Aghia Paraskevi Athens, Greece; 4 Department of Environmental Modelling, Institute of Mathematical Machine and Systems Problems, National Academy of Sciences of Ukraine, Kiev, Ukraine

## Abstract

The intentional or accidental release of airborne toxics poses great risk to the public health. During these incidents, the greatest factor of uncertainty is related to the location and rate of released substance, therefore, an information of high importance for emergency preparedness and response plans. A novel computational algorithm is proposed to estimate, efficiently, the location and release rate of an airborne toxic substance source based on health effects observations; data that can be readily available, in a real accident, contrary to actual measurements. The algorithm is demonstrated by deploying a semi-empirical dispersion model and Monte Carlo sampling on a simplified scenario. Input data are collected at varying receptor points for toxics concentrations (*C*; standard approach) and two new types: toxic load (*TL*) and health effects (*HE*; four levels). Estimated source characteristics are compared with scenario values. The use of *TL* required the least number of receptor points to estimate the release rate, and demonstrated the highest probability (>90%). *HE* required more receptor points, than *C*, but with lesser deviations while probability was comparable, if not better. Finally, the algorithm assessed very accurately the source location when using *C* and *TL* with comparable confidence, but *HE* demonstrated significantly lower confidence.

## Introduction

In modern societies, where risk has a dominant role in all facets of our life^1,2^, short-term exposure to toxic/hazardous material (HazMat), whether intentional or accidental, poses great threats to the surrounding population^3–5^. The Sarin gas terrorist attacks in Japan (Matsumoto^6^ and Tokyo^7^), as well as the famous chemical accidents of Bhopal, India^8^ and Seveso, Italy^9^ are characteristic examples of such incidents.

An unlikely event of a toxic/hazardous material begins with a “source” releasing the HazMat in the air, depending on the meteorological conditions, the plume is dispersed reaching, potentially, high HazMat “concentrations” that vary over space and time. A subject’s (receptor’s) “exposure” to the HazMat depends mainly on the subjects’ activity and residence time within the plume. The amount - “dose” - deposited within the body is affected by a number of biological factors. Finally, the subject’s “response” to the HazMat event will be exhibited by a series of adaptive or adverse health effects. This is the consequences analysis paradigm^10^ in which the final impact of a HazMat release depends on the “source-concentration-exposure-dose-response” binary relationships. However, among the required information for emergency response planning, the source characteristics present the greatest factor of uncertainty^11^ and can differ from the true one by a factor of 10 or more^12^. Thus, the identification of source characteristics is of high importance for assessing the health impact on the population and planning exposure measures for epidemiological studies^13^ covering all environmental compartments^14^.

The reconstruction of the source term of an airborne contaminant may be obtained by using forward (optimization/minimization) or backward (inverse) approaches, in which source characteristics are inferred from concentration or deposition measurements at different locations and time intervals by establishing source-receptor (i.e. source-concentration) relationships^15^. The problem itself is not new. It is driven mainly by the investigation of nuclear-energy accidents in the global^16^ or local scale^17^ and it is not limited to airborne contaminants^18^. The first class of methods includes Gradient based techniques (e.g. least squares^19^, re-normalisation^20^ and Broyden-Fletcher-Goldfarb-Shanno algorithm^21^), Meta-heuristics (e.g. pattern search method^22^, simulated annealing^23^ and genetic algorithms^24^), Bayesian inference approaches^25,26^ and Markov Chain Monte Carlo (MCMC) sampling techniques^27,28^. More details for the methods of first category may be found in the recent review paper by Hutchinson *et al*.^29^. The second type of methods incorporates adjoint and tangent linear models^30–32^, Kalman filtering^33^ along with Gaussian^34^, Lagrangian^35,36^ and advanced dispersion models^28,37^, as well as variational data assimilation techniques^38,39^. In any case, the greater the number of available concentration measurements, the closer the guessed/estimated source rate will be to reality, resulting to more reliable and faster mitigation. For example, Say, *et al*.^40^ inferred multi-annual UK emissions of Hydrofluorocarbons and Schauberger, *et al*.^41^ completed a fragmented set of emission rates from a wastewater treatment, both by using the respective atmospheric observations. Unfortunately, concentration measurements are rarely available on-site unless for a high-risk facility or a regional-scale accident.

The concept of information-reconstruction has also been employed on the third and fourth relationships (exposure-dose and dose-response), of the consequences paradigm i.e. the use of available dose or response data to estimate the exposure levels. However, no source reconstruction has been attempted, because the aforementioned source reconstruction models take into account only concentration data^42^. For example, Pirkle, *et al*.^43^ used biomonitoring data, such as clinical tests and biomarkers, in order to identify lead exposures, while Hays, *et al*.^44^ discussed issues inherent in using clinical tests for evaluating such data. Other researchers^45^ moved one step further by using Physiologically Based Toxicokinetic (PBTK) and Biologically Based Dose-Response (BBDR) models, together with appropriate optimisation and inverse modelling techniques to reconstruct exposure to environmental chemicals, and to some extent the source itself, from biomarkers. PBTK models approximate the kinetic behaviour of chemicals and, as a result, can predict the internal dose at targeted tissues/organs^46^. On the other side, BBDR models represent biological processes at the molecular and cellular level which link the target issue/organ dosage to the health outcome^47,48^. Later, Chen, *et al*.^49,50^ employed chemical data (i.e. blood sampling and urinary data) in conjunction with Monte-Carlo sampling techniques for reconstructing past exposures. In the same direction, simplified PBTK models and clinical data were deployed to demonstrate the reconstruction of exposures, to BisPhenol A^51^ and Carbaryl^52^.

In all these studies, the main objective was to estimate (reconstruct) exposure to a chemical(s) by using primarily clinical tests and biomarkers. Whereas, only a few of them attempted to incorporate the observed health effects (clinical health observations e.g. asthma, gastrointestinal problems, and deaths) which are among the first type of information collected by the response personnel. Therefore, this study demonstrates the concept of reconstructing source characteristics based on health observations in the case of acute chemical, biological or radiological accidents. A simplified forward-modelling source-reconstruction algorithm was developed based on the SLAB semi-empirical dispersion model^53^ and the Monte Carlo sampling technique. A realistic toxic gas release scenario (synthetic scenario) is presented to assess the performance and capability of the proposed concept. To the authors’ best knowledge, this is the first report of this concept.

## Methods

**Figure 1:**
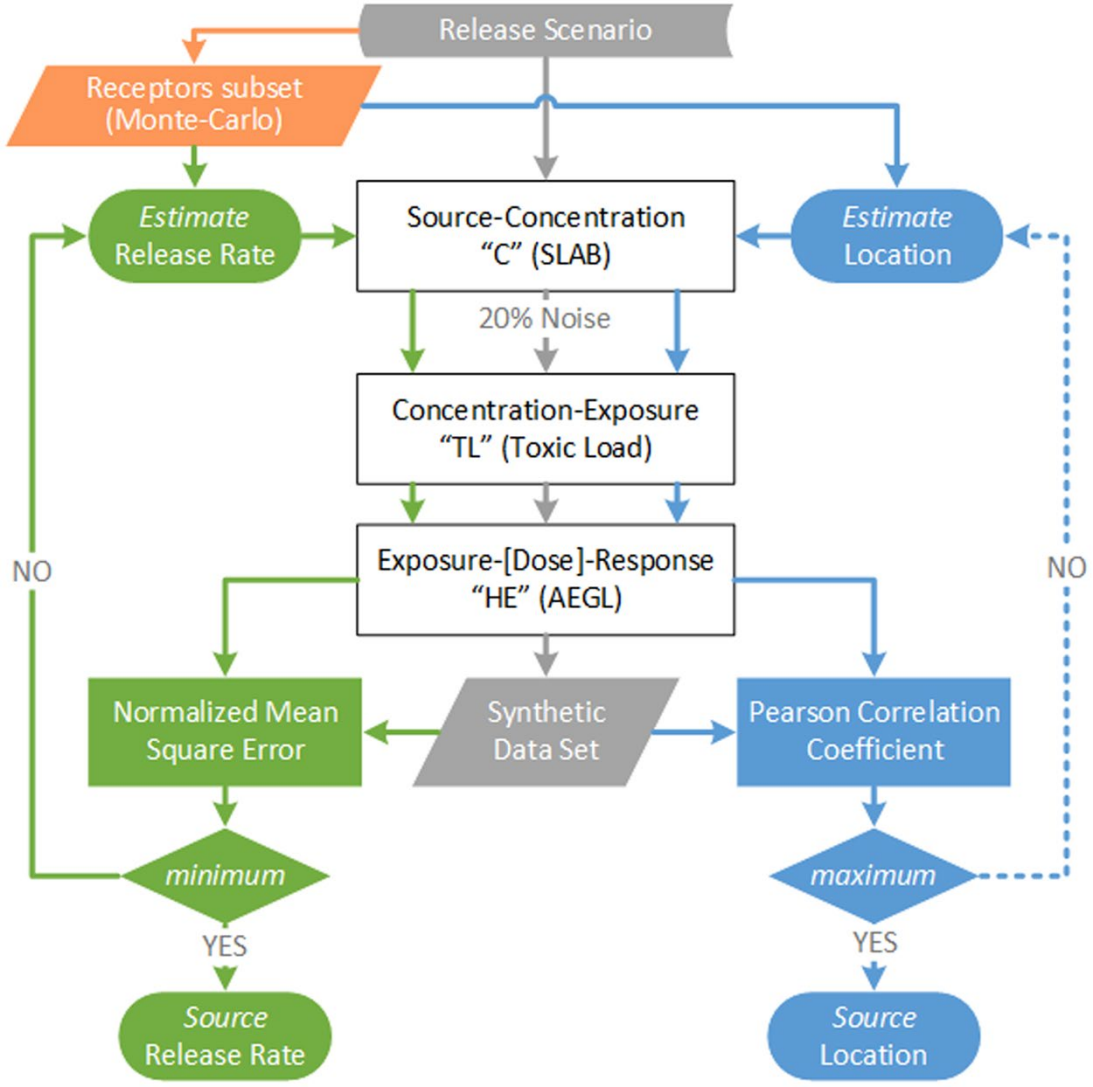
Outline of the developed computational algorithm and presented methodology.

In the absence of any similar previous work, it is first necessary to identify the expectations and limitations of such an algorithm. In principle, the algorithm should be able to combine available response data to estimate the source characteristics. Typically, such a problem can be solved by either optimizing the forward solution or solving the inverse mathematical equation^15^ e.g. advection equation. However, there is no single equation that relates source-response directly, instead there are four different sets of equations that cover the binary relationships of source-concentration, concentration-exposure, exposure-dose, and dose-response. Often, some of these sets of expressions are based on stochastic and probabilistic approaches. Moreover, response data, collected by emergency response teams, will usually be in the form of discrete information (e.g. clinical health effects). Following the above reasoning, it is evident that the proposed concept includes a number of inherent difficulties, although promising and attractive, and the use of an advanced inverse numerical scheme could further increase the complexity. Therefore, the first version of the algorithm was designed using simplified steps and approaches, in order to be able to investigate the proposed concept and assess its performance. The overall outline of the methodology is presented (Fig. 1).

First, a simplified and realistic release scenario is formulated and forward modelling is implemented at three steps (Fig. 1, white blocks). The results of the forward modelling comprise the synthetic dataset which plays the role of real data collected by the response teams and will be used as input for the source reconstruction algorithm. Note that a random numerical noise in the range of ±20% has been added to the concentration values (only at the forward atmospheric dispersion modelling results) to represent the expected uncertainty of real measurements and to account for the input and meteorological conditions variability. The selection of the noise range is based on the recent findings of a related EU project^54^.

It should be noted that the meteorological data for the source reconstruction can be a difficult problem to address due to the lack of reliable data. In order to surpass this problem a number of researchers has proposed some interesting techniques for increasing the confidence of the selected meteorological data. For example, Allen *et al*.^55^ attempted to determine directly by their proposed algorithm the surface wind direction along with the reconstruction of the source characteristics. Kolczynski *et al*.^56^ performed ensemble forecasting in order to quantify the uncertainty of the meteorological data. They used multiple numerical weather prediction models adopting different initial and boundary conditions for each model configuration. Another interesting approach to treat the meteorological data uncertainty by Zajaczkowski *et al*.^57^ is the combination of numerical weather predictions with advanced CFD techniques for the wind prediction. More specifically, the method assimilates mesoscale model data (e.g. wind profiles) into the selected CFD model presenting improved accuracy. In the present study, we used available meteorological data from an airport location, however, in a future research we will examine the proposed algorithm with the assimilation of weather data into the ADREA CFD code^58,59^.

Then, a sample (receptors subset) of the synthetic dataset is selected via Monte-Carlo sampling, in order to be used as input parameter into the source reconstruction algorithm. The release rate estimation and location identification take place in two different, parallel, and loop processes, each using the forward modelling scheme until the set goal is achieved (Fig. 1 green and blue blocks).

Finally, it is important to mention that the release rate and location of the source may also be calculated based on the mean square error, so it is not necessary to estimate location separately, as we shown in Fig. 1. However, we did not select this approach because there is a numerical advantage regarding the convergence of the solution, as shown in our recent work by Efthimiou *et al*.^58^.

### Description of the release scenario

The considered scenario involves a release from a feed pipeline of natural gas with a source rate of 100 kg s^−1^ and leak duration of 10 min. The gas contains 0.77% (w/w) of hydrogen sulphide (H_2_S), a well-studied toxic agent with adverse health effects even at sub-ppm concentrations^60,61^. The release of H_2_S is directed to the non-process building, while the temperature and pressure of pipeline are assumed to be 27 °C and 83 bars, respectively. The scenario is based on an own previous study^62^ and a related Quantitative Risk Assessment (not publically available). The surrounding area of the facility is characterised as flat without obstacles. The prevailing wind speed during the release is taken equal to 5 m s^−1^, while a class *D* to “(neutral conditions) atmospheric stability has been selected.

A computational domain area of 5000 × 1000 m^2^ was selected for the numerical simulations. Then, the domain area was divided into a 200 × 40 grid, with *Δx* = *Δy* = 25 m. The coordinates of the actual source location were chosen at (x, y, z) = (500, 300, 0), while all the source parameters were assumed to be constant over the time. The same grid was used to identify possible source locations.

### Source-concentration modelling

The SLAB atmospheric dispersion model was selected to simulate the H_2_S plume and predict the concentration levels with respect to time at each grid node (x and y coordinates) and at height 2 m above the ground (selected breathing zone). The SLAB model was developed by Ermak and co-workers^53,63^ and is suitable for continuous and finite duration release scenarios^64^. It has been extensively validated against lab- and field- experiments, presenting satisfactory agreement^53^.

### Concentration-exposure and exposure-dose-response modelling

The contact with a toxic agent through ingestion, inhalation and dermal route can lead to adverse health effects due to the absorption or adsorption of the material from the human body. This forms a dose and potentially turns out a disease issue^42^. Thus, depending on the toxic agent characteristics (i.e. nature and concentrations), the impact of the agent can vary significantly, while the duration of the exposure at low concentrations can be extended for a longer period^65^.

The impact of exposure is estimated by using three acute exposure guideline levels (AEGLs)^66^. The AEGL thresholds determine the exposures (i.e. constant concentrations levels) for given durations from 10 min to 8 hrs, presenting three levels of harm or the general public, namely notable discomfort (AEGL-1), disability (AEGL-2) and life threatening or death (AEGL-3)^66,67^. However, in a real release scenario, inhalation dosages may vary significantly with durations, which pose a challenge to estimate the onset of the effects using the fixed values by AEGLs.

To surpass this problem, a toxic load algorithm, based on the AEGLs and quantification of the toxic dosage, using a non-linear equation^68^, was proposed^69^. When this toxic dosage exceeds a value of one, then the respective AEGL threshold has been reached. By adopting this approach, we computed the onset of each of the AEGL thresholds either as a continuous toxic load (i.e. *TL*) or as discrete health effects (i.e. *HE*, discrete values of AEGL-1, AEGL-2, AEGL-3, and “nothing”) for every available concentration profile. The *TL* predictions require the AEGL thresholds for the considered chemical (i.e. for H_2_S see Table 1). A power law of 4.4 is obtained by fitting the AEGL data of Table 1 (R^2^ better than 0.99), while we define a low and upper bound of exposure duration equal to 30 secs and 24 hrs, respectively.

**Table 1:** Acute exposure guideline levels (AEGLs) for H_2_S (https://www.epa.gov/aegl).

Exposure duration	10 min	30 min	1 h	4 h	8 h
**H**_2_ **S (ppm)**					
AEGL-1	0.75	0.60	0.51	0.36	0.33
AEGL-2	41	32	27	20	17
AEGL-3	76	59	50	37	31

The proposed algorithm “EAGLE”^69^ is based on an application of the “Induction Parameter Model”^70^ which employs a non-linear equation^68^ in order to quantify the toxic dosage^71^. EAGLE is also capable of coupling with a dispersion model in order to enable the prediction of the onset of AEGLs thresholds for time-varying plume involving chemical agents with tabulated AEGLs. The approach can be determined by the following expressions:

1 $$TL(t)={\int }_{0}^{t}T{L}_{rate}({t}^{\text{'}})d{t}^{\text{'}}$$

2 $$T{L}_{rate}(t)\,:=\frac{dTL}{dt}=\frac{1}{{t}_{b}}{[\frac{C(t)}{{C}_{{t}_{b}}}]}^{n}$$

where *TL (t)* is the integrated toxic load, *t* is the time, *t*_*b*_ is the reference AEGL time band exposure step, ${C}_{{t}_{b}}$ is the concentration corresponds to the reference AEGL time band exposure time (Table 1) and n (=−0.23 for H_2_S) is the power exponent. In this work, the EAGLE algorithm was programmed to evaluate the toxic load (exposure) and the corresponding health effects (response) resulting from the estimated H_2_S concentration levels

### Source reconstruction and Monte Carlo sampling

In order to obtain an optimal estimate of the source rate (*Q*_*s*_), we adopted an optimisation routine that is based on minimising a cost function, namely normalised mean square error:

3 $$NMSE=\frac{\langle ({C}_{m}-{C}_{o})\rangle }{(\langle {C}_{m}\rangle -\langle {C}_{o}\rangle )}\to min$$

where triangle brackets denote arithmetic averaging, while *m* and *o* subscripts stand for model and observations, respectively. The iterative calculation was performed by the “fminbnd” built-in function of MATLAB R2016a;an algorithm based on golden section search and parabolic interpolation^72,73^.

The identification of the release location can be obtained from another optimisation routine that is based on maximizing a cost function based on the Pearson correlation coefficient, *J*^74^:

4 $$J=\frac{1}{N-1}\sum _{i=1}^{N}(\frac{{C}_{o,i}-\langle {C}_{o}\rangle }{{\sigma }_{o}})(\frac{{C}_{m,i}-\langle {C}_{m}\rangle }{{\sigma }_{m}})\to 1$$

where *C* is the concentration and *σ* is the standard deviation. *J* approaches a value of unit for two perfectly correlated sets of observations and modelling results. Since the source rate does not directly participate in the correlation coefficient, the obtained solution is independent of the choice of the source rate^58,75^. The source location could be calculated with a multidimensional optimisation function, but in our case for illustrative reasons we compute *J* (Eq. (4)) at all possible grid points (receptor points) because of the small computational demand.

Monte Carlo simulations are also performed to generate multiple samples of varying size of observations. Hereafter, we refer to the size of the observations sample as number of receptors (ranging from 2 to 100) and the number of samples as the number of iterations (10, 100, 1000, and 10000). In other words, each iteration is comprised by a predefined number of randomly selected receptors. The receptors’ network or distribution is an important aspect of source reconstruction and this is captured by studying multiple and varying number of iterations. However, the effect of the receptors’ position and other specific characteristics have not been studied explicitly. Finally, the sampled results (subset of the synthetic dataset) are then imported into the reconstruction algorithm.

## Results

This section presents the results of the source reconstruction algorithm i.e. performance towards estimating the correct source release rate and location. It also includes results of the algorithm sensitivity against the two most important parameters, the number of receptors and number of iterations. The time period at which data are collected, although critical in a real incident, did not affect the behavior of the algorithm. Therefore, here only results for the first 600 s after the incident are presented.

SLAB computation times are in the scale of <1 s per simulation. Our inverse modelling approach scales linearly with the number of available receptor points, in practice less than 20–40 receptors. Overall computation time was less than 1 min per iteration (i.e. specific group of receptors). We used a multi-CPU system in order to conduct the multiple-iterations studies.

### Prediction of source rate (Q_s_)

**Figure 2:**
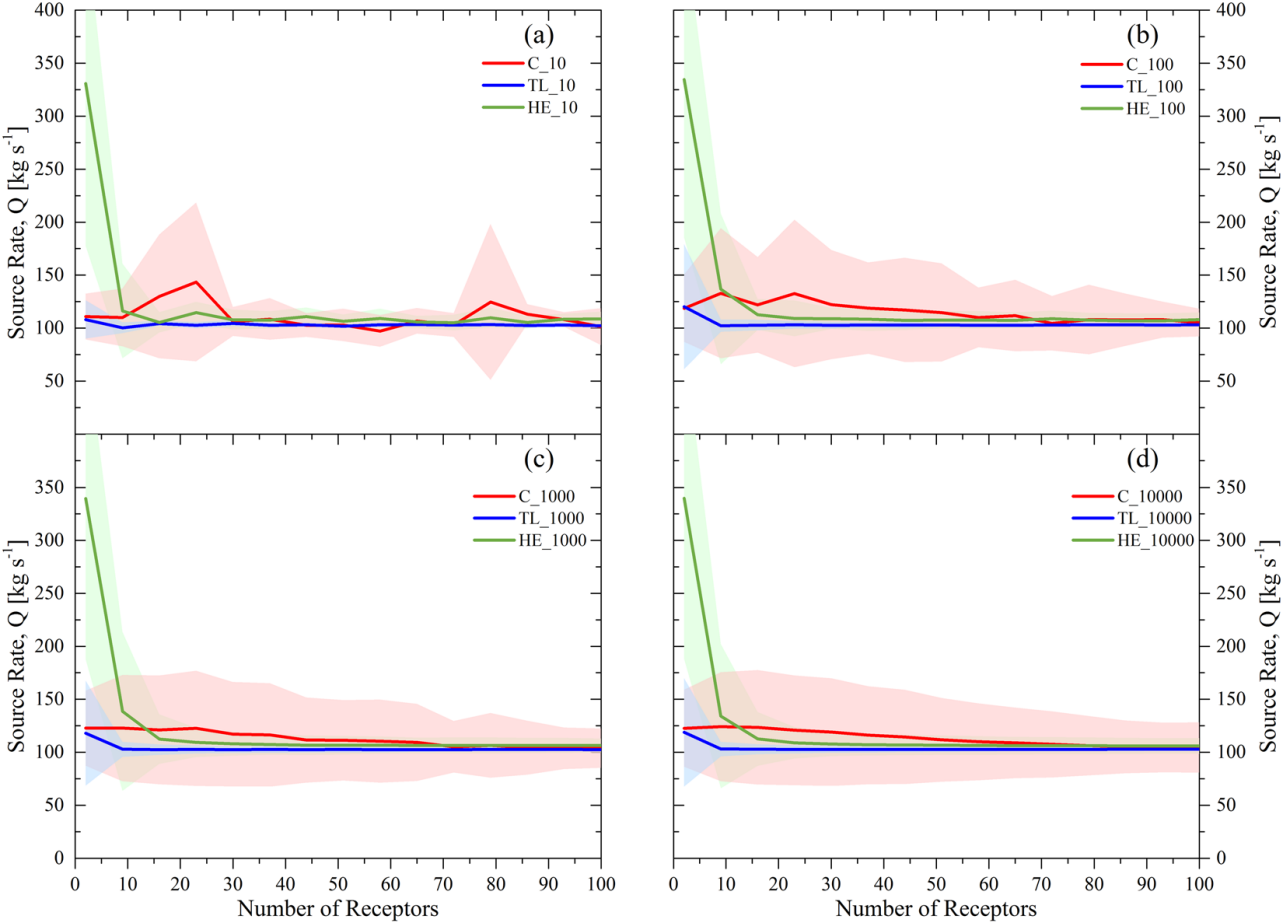
Estimation of source rate value according to the selected number of receptors: (**a**) 10 iterations, (**b**) 100 iterations, (**c**) 1000 iteration and (**d**) 10000 iterations, for Concentration (*C*; ), Toxic Load (*TL*; ) and Health Effects (*HE*; ) considered parameters. The three shaded areas represent the 2σ range for *C* (red shaded area), *TL* (blue shaded area) and *HE* (green shaded area), respectively.

Figure 2(a–d) exhibit the estimated source rate based on the different number of receptors ranging from 2 to 100 sample points. The proposed model was run for four number of iterations (i.e. 10, 100, 1000 and 10000) in order to investigate the impact of increasing the magnitude on the convergence of the results. We observe that in any case of chosen number of iterations the estimated source rate approaches sooner/faster the value of the real one (~100 kg.s^−1^) when the dataset is obtained from the *TL* (solid blue line). Accordingly, the *2σ* range of *TL* (blue shaded area) is also smaller than *C* (red shaded area) and *HE* (green shaded area) methods.

By using the *HE* method (green solid line), we notice that there is a delay to approach the real value and a larger scattering area (green shaded area) compared to the other two methods. The use of *C* values as input data in the source reconstruction algorithm gives better convergence than *HE* dataset but worse than *TL*.

**Figure 3:**
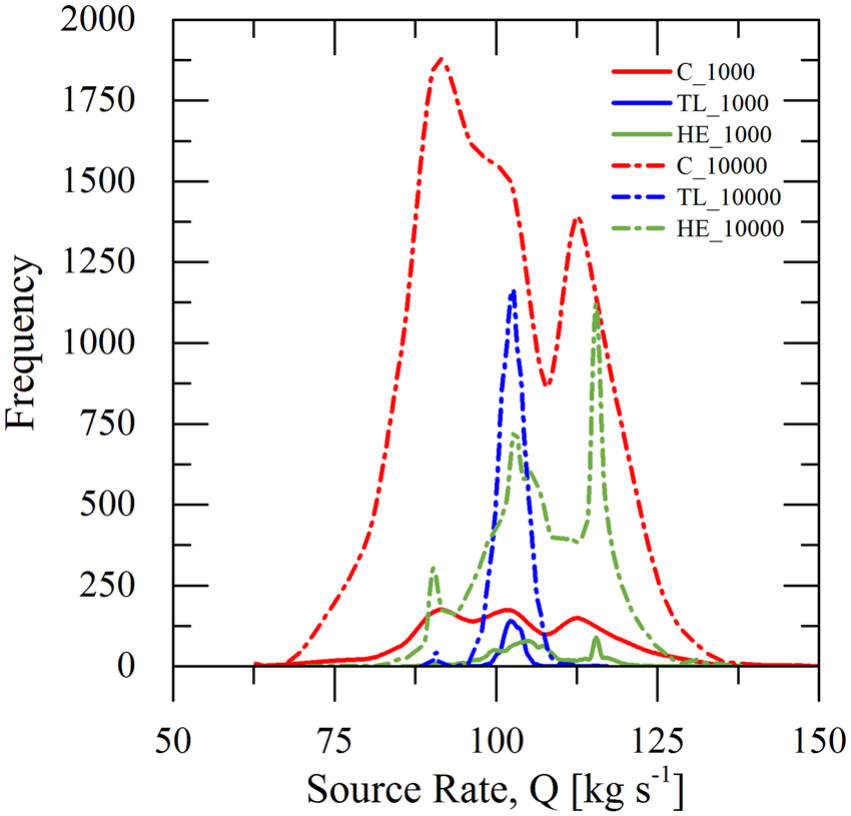
Values of number of occurrences versus the source rate estimation of 1000 (solid line) and 10000 (short dash dot line) iterations, respectively, for Concentration (*C*; red colour), Toxic Load (*TL*; blue colour) and Health Effects (*HE*; green colour) considered parameters. The number of selected receptors is taken equal to 100.

Figure 3 illustrates the number of occurrences versus the estimated values of source rate for 100 receptors and two number of iterations (1000 and 10000). The frequency values for the estimated values of source rate were calculated by using input data of *C*, *TL* and *HE*. The estimated results for the source rate after 1000 iterations lie between 62.8 and 130 kg s^−1^ for *C* method, from 90.26 to 115.74 kg s^−1^ for *TL* method, and 87.5 − 113.5 kg s^−1^ for *HE* method. Note that the smaller the obtained surface from the curves at each selected number of iterations, the higher the portion of estimates being close to the real solution. Similar results, we can observe for 10000 iterations, however, the number of occurrences is much higher than in the case of 1000 iterations.

**Figure 4:**
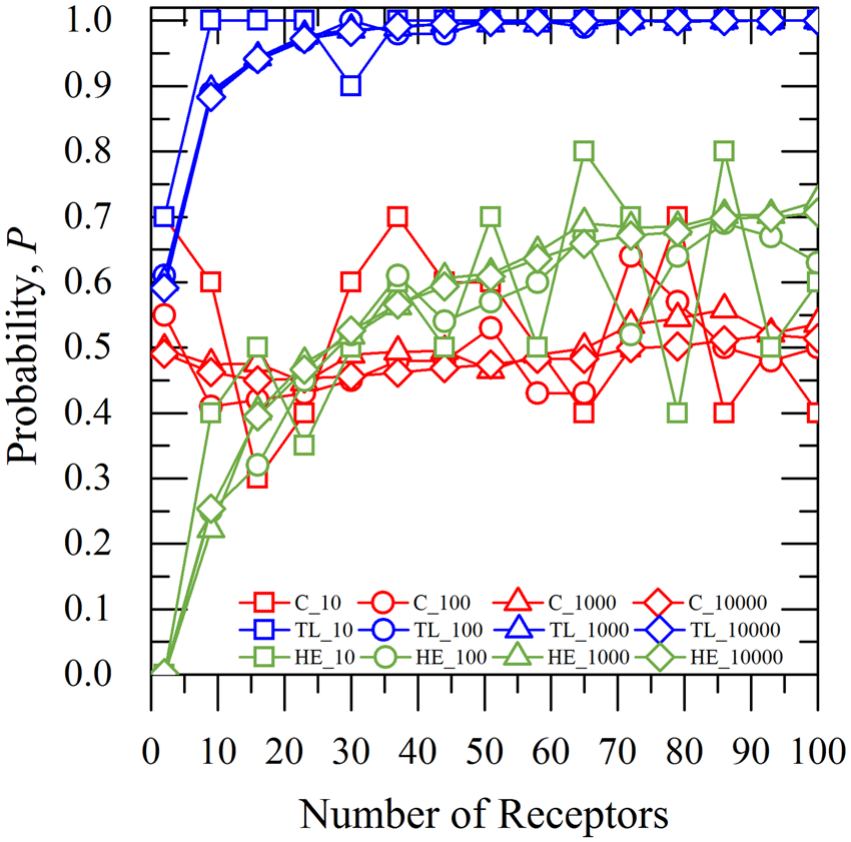
Values of Probability factor depending on the selected number of receptors. Number of 10 (□), 100 (○), 1000 (∆) and 10000 (◊) iterations, respectively, for Concentration (*C*; ), Toxic Load (*TL*; ) and Health Effects (*HE*; ) considered parameters.

Another important factor of assessing our results is to compute the probability of the estimated values of source rate to approach the real value versus the selected number of receptors (sample points)^32^, as shown in Fig. 4. As the probability values approach one, the estimated value of source rate is close to real source rate. From Fig. 4, it is also seen that the highest probability values are obtained from the *TL* method, which after a certain number of receptors (~40) approaches one, regardless of the selected number of iterations. For *C* dataset, we observe that the probability values are close to 0.5 for more than 50 receptors and large number of iterations (i.e. 1000 (red triangle symbol) and 10000 (red diamond symbol). For *HE*, we notice that the probability of the solution approaches 0.7 for more than 60 receptors and in some cases (green square symbol) overpasses it even with relatively small number of iterations (i.e. 10). It is also indicated that the probability of the solution for *HE* is better than *C* for more than 25 receptors. It is worth mentioning that the increased number of iterations also helps to achieve faster convergence of the results.

On the other hand, the probability values for the estimated source rate are gradually decreasing for less than 50 receptors, however, in the case of *C* the decrease of the obtained probability is almost negligible for 100 (red circle symbol), 1000 (red triangle symbol) and 10000 (red diamond symbol) iterations, excluding the case of 10 (red square symbol) iterations.

### Prediction of source location

The location of the source release was predicted using three different input datasets (*C*, *TL* and *HE*), and each time the number of receptors was varied between 2 and 100, for four different number of iterations (10, 100, 1000 and 10000). The present results assess the performance of the proposed algorithm based on the different types of input data and number of receptors required in order to obtain the desired results (source location).

**Figure 5:**
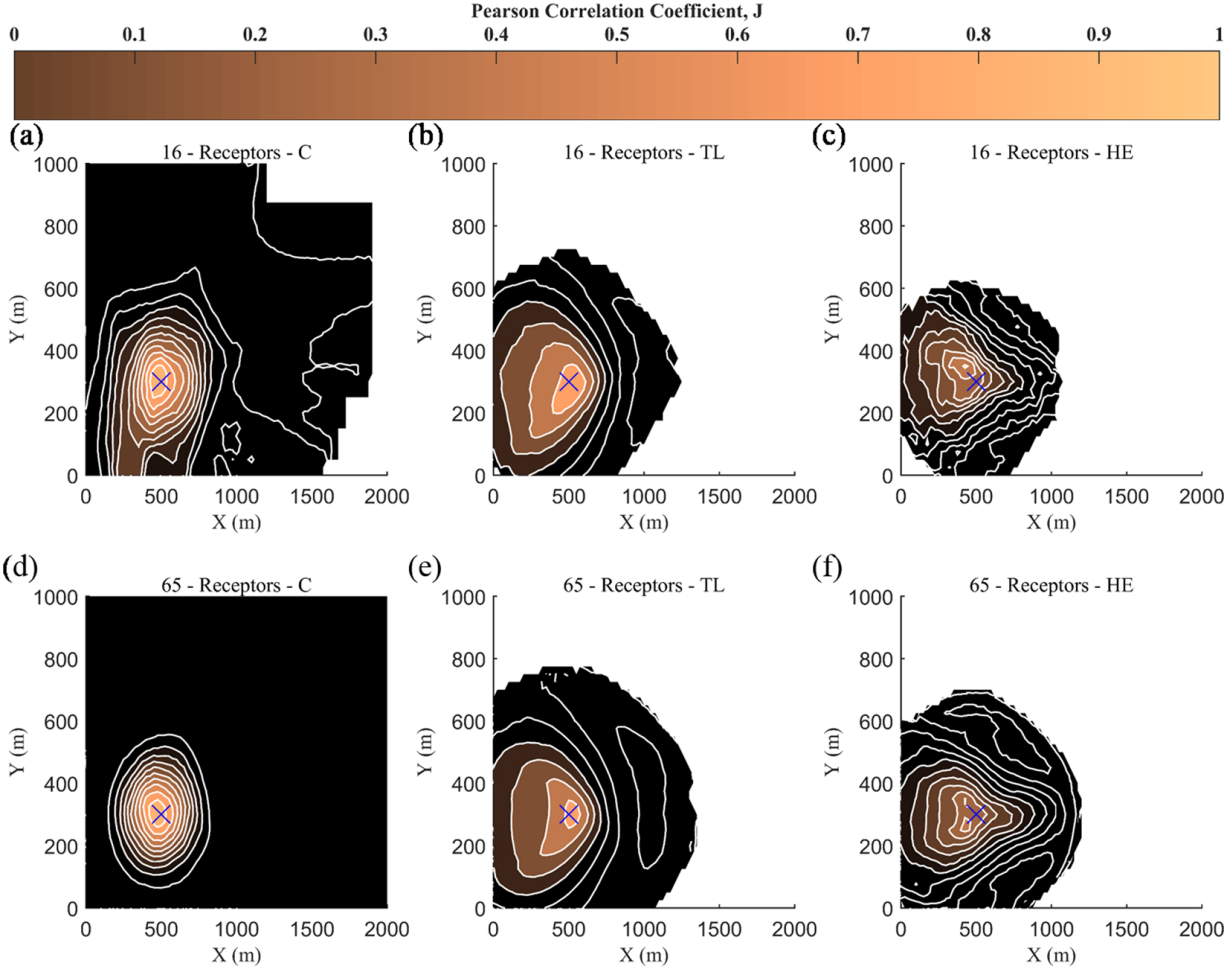
Spatial distribution of the correlation coefficient, *J*, for 16 (**a**–**c**) and 65 (**d**–**f**) receptors used for values of Concentration (**a**,**d**), Toxic Load (**b**,**e**) and Health Effects (**c**,**f**). The blue cross symbol denotes the real source location and the selected number of random iterations is taken equal to 100.

Figure 5 presents the spatial distribution of the correlation coefficient (*J*) corresponding to each guessed source location coordinates, for 16 and 65 receptors with input data from *C*, *TL* and *HE* and for 100 iterations. The colour bar depicts the colours that correspond to the values of Pearson Correlation Coefficient (PCC). Values close to one indicate that the solution is approaching the real release location (blue cross symbol). The prediction of source location is well identified, in all cases, regardless of the low *J* values for *HE*. This significant outcome indicates the advantages of the *J* compared to the NMSE for the source location estimation. As a result, we can claim that the source location is estimated with high accuracy when *C* or *TL* are available, and high probability when *HE* is adopted.

**Figure 6:**
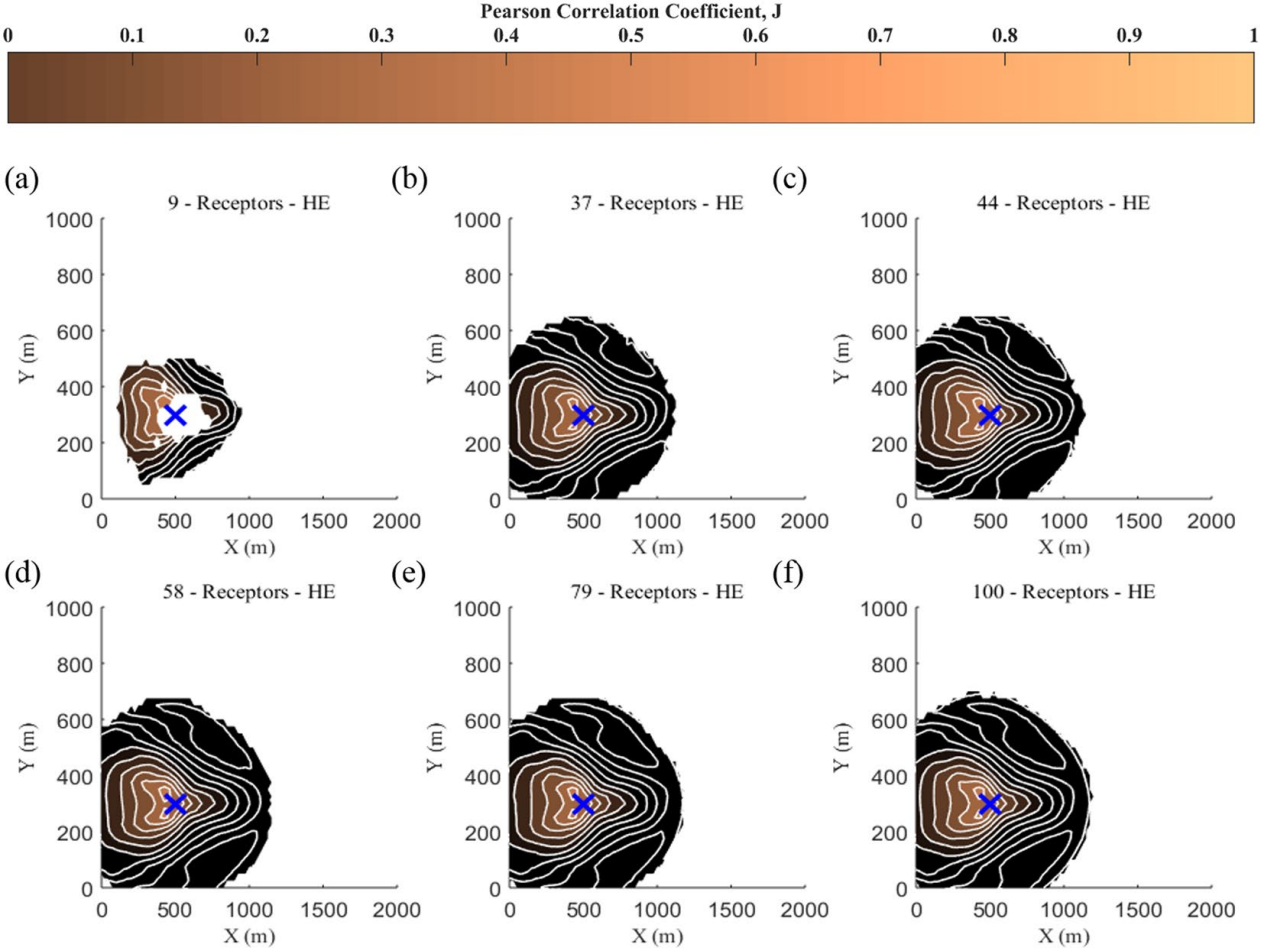
Spatial distribution of the correlation coefficient, *J*, for health effects observations and 100 iterations using (**a**) 16, (**b**) 37, (**c**) 44, (**d**) 58, (**e**) 79 and (**f**) 100 receptors. The blue cross symbol denotes the real source location.

Figure 6 depicts the results for the prediction of source location using as input data health effect observations and 100 iterations. It is seen that the level of the complexity associated with the use of health effects as input parameter has effect on the minimum required number of receptors for the source location reconstruction. It is also noticed that after 58 receptors the reconstruction of source location is well predicted, but the required number of receptors for the desired results is larger than *C* and *TL* scenarios.

**Figure 7:**
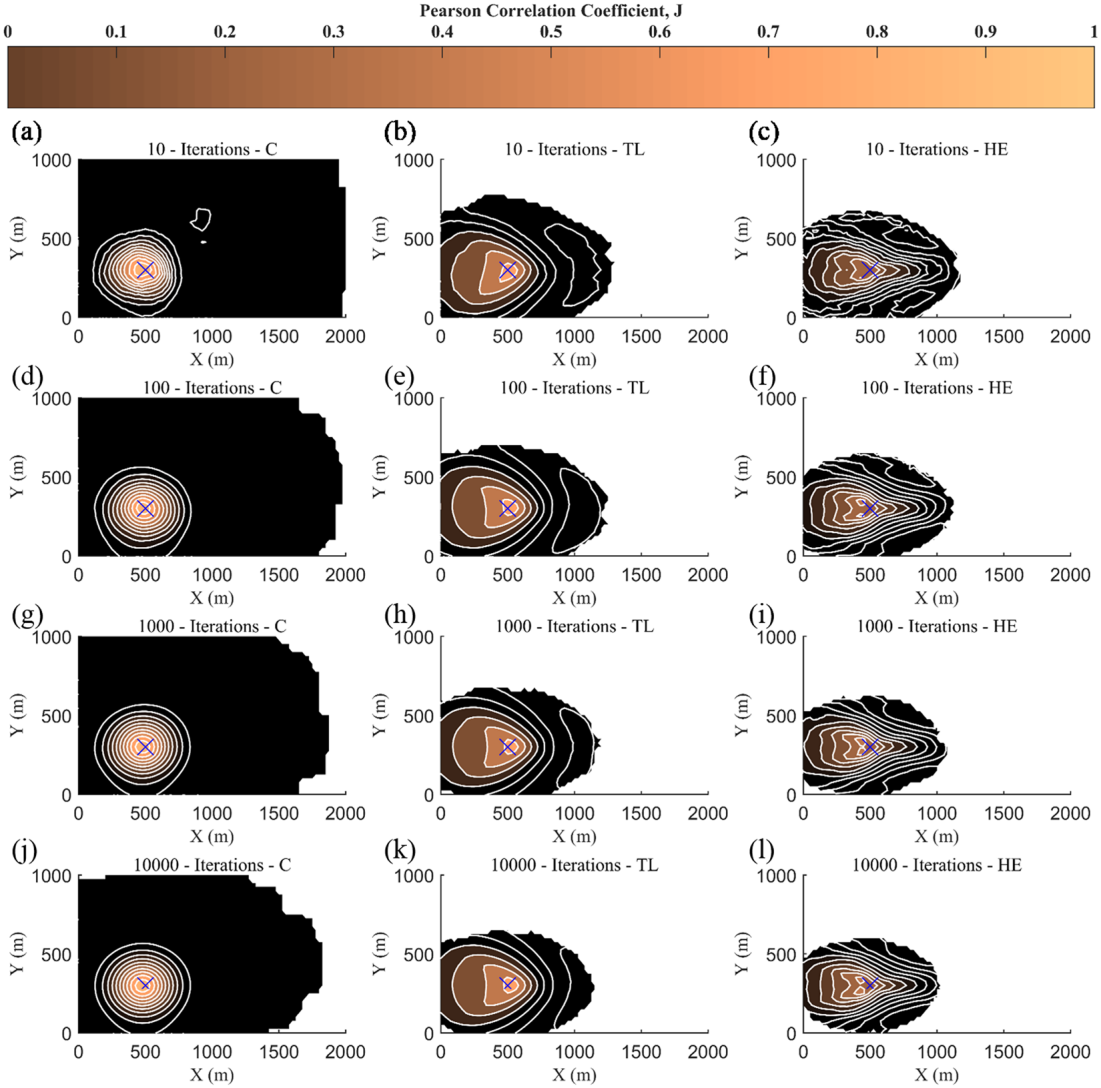
Spatial distribution of the correlation coefficient, J, for values of Concentration (**a**,**d**,**g**,**j**), Toxic Load (**b**,**e**,**h**,**k**) and Health Effects (**c**,**f**,**i**,**l**) using 10 iterations (**a**,**b**,**c**), 100 iterations (**d**,**e**,**f**), 1000 iterations (**g**,**h**,**i**) and 10000 iterations (**j**,**k**,**l**). The used number of receptors is 37. The blue cross symbol denotes the real source location.

According to Fig. 7, it is observed that with increasing the number of iterations the results are converged faster and the obtained source location approaches the real one (blue cross symbol). It is also worth mentioning that even with small number of iterations and relatively small number of receptors (i.e. 37) the source location is well retrieved, in particular for the cases of *C* and *TL* input data.

**Figure 8:**
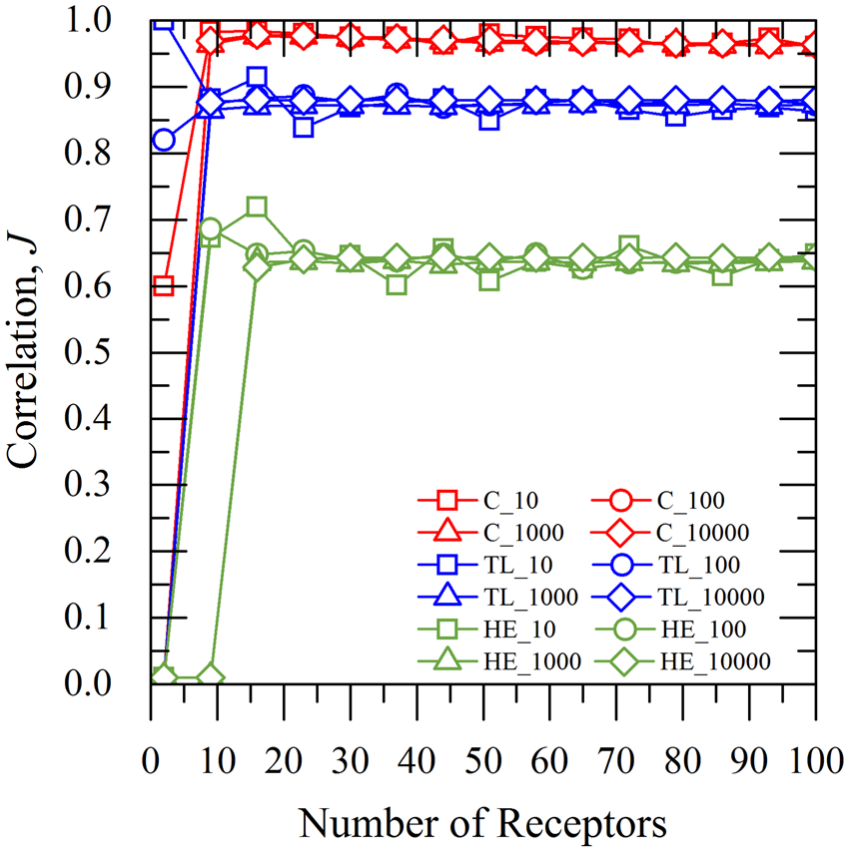
Values of correlation factor depending on the selected number of receptors. Number of 10 (□), 100 (○), 1000 (∆) and 10000 (◊) iterations, respectively, for Concentration (C; ), Toxic Load (TL; ) and Health Effects (HE; ) considered parameters.

Figure 8 depicts the dependence of the correlation coefficient (*J*) over the selected number of receptors. The obtained results are derived from 10, 100, 1000 and 10000 iterations for the corresponding *C*, *TL* and *HE* input. The correlation coefficient approaches the highest values around 0.95 for *C* (red solid line), after a certain number of receptors (less than 10) regardless of the set of iterations. Similar behaviour is observed for *TL* but the values of *J* are lower-around 0.8. On the other hand, the lowest values of *J* (~0.65) are obtained from the *HE* (green solid line), as shown in Fig. 8. In any case, uncertainty of the source location proves to be independent of the number of receptors and iterations for any test past the 10–20 receptors. In other words, a small number of receptors yields satisfactory values for *J*, as less as 10 receptors. It is also worth mentioning that no value for the source rate is necessary.

## Discussion

The sensitivity of the numerical results to the selected input data was gauged by varying the number of receptor points used in each scenario individually. With respect to the performance of the proposed scheme in reconstructing the source rate, the obtained information from the three considered methods (*C*, *TL* and *HE*) allowed the prediction of “averaged” source term rate in the range of 45–450 kg s^−1^. Therefore, a wide variation in the quality of the algorithm’s prediction was observed, in particular for the use of *HE* dataset as input parameter.

Our results show that the use of *C* data along with the increase of sample points used in reconstructing the release source rate leads to better convergence and agreement of the obtained results with the actual release source rate. The necessity to have large number of input data for the source reconstruction algorithm is due to the inherent variability and uncertainty of predicting the real release rate. Herein, this is reflected mostly with the introduced noise to the synthetic data, as discussed in Section 2. With the absence of artificial noise, this is a simple well-posed problem at which the obtained solution algorithm converges in few iterations together with a small number of receptors. However, in reality, uncertainty is high and only scarce number of data are typically available for use in the source rate estimation, thus the examined scenarios are only for exploratory purposes aimed primarily at assessing the limitations associated with the proposed computational framework.

By using the averaged source rate predictions obtained from the *TL* approach, it is seen that less variability and good agreement is achieved, when utilizing 10 receptors points and above (Fig. 2). The results are also independent for a selected number of 51 receptors and above. The different performance of the proposed reconstruction algorithm between *C* and *TL* input data can be attributed to the fact that the *TL* is estimated using an integral of time and hence it contains prior information (history) of the specified period at which the data are collected. With the use of *HE* as input parameter the performance of the method also improves with significantly reduced variability compared to the *C* input data. On the other hand, the prediction of the source location, using *ΗΕ* data, requires twice as much information (receptors and iterations) in order to achieve a comparable estimate as in the case of *C* and *TL* data (Fig. 7).

Another parameter that influences the performance of the algorithm and quality of the results is the number of iterations used in generating the source rate predictions. By increasing the number of iterations is shown to yield better convergence and less spread of the results around the average. This can be attributed to the use of large number of iterations, which accounts for different possible combinations of a certain number of receptors rather than only a single dataset of a given number of receptors. During realistic conditions, however, the errors of the obtained solutions are likely to appear within the confidence limits, which were estimated using the large number of iterations. It should also be noted that in realistic conditions the errors of model predictions might be larger than those generated in simulation exercises. Therefore, the confidence intervals may be also larger. The use of random combination herein is for exploratory purposes to assess the performance of the algorithm against the uncertainty related to the use of sets of points with the same size but collected at different locations.

Nonetheless, there is room for future work in order to increase the accuracy of the results. Such as the use of more advanced dose-response models (e.g. PBTK), inclusion of variable meteorological data and site topography (e.g. CFD models) and more complex information related to the toxic release (e.g. used in asymmetric attacks or transport accidents, degradation of agents^76^, and multiple agents/sources^77^). Overall uncertainty could be reduced by the utilization of other statistical tools like Markov chain Monte Carlo, Bayesian inference, and clustering to determine the probability of the solution^22^. Alternatively, the implementation of more advanced numerical algorithms could partially overcome the limitations of the simple dispersion model. Ma and Zhang^78^ improved the source parameters estimation, of a simple Gaussian model, by combining an integrated Gaussian-Machine Learning model with the particle swarm optimization approach and later^79^ introducing the Tikhonov regularization approach. Further improvement and increasing of the accuracy of the results may be gained by including in the formulation observations from multiple time periods, instead of a single time-integration (dose) per location (sampling point) as it was demonstrated in other cases^80^ and herein. Finally, pre-calculated response (health impact) databases could be combined with stochastic methods such as artificial neural networks to develop response-source correlations which then could be used with real-time observations to reconstruct the source-parameters, similarly to dispersion pattern recreation without the source knowledge^81^.

## Conclusions

The work described herein focuses on the prediction of the released amount of a hazardous substance and release location using health observations instead of concentration levels (standard approach) or dosages is some cases. The proposed methodology is based on the coupling of multiple methods to resolve the source-concentration-exposure-dose-response binary relationships and fully elaborate the consequences analysis paradigm.

The results present good predictions of the source characteristics using the health observations as input for the proposed source reconstruction algorithm. The accuracy of the method, in reconstructing the source parameters decreases when a small set of observations or input data are available. Nonetheless, it is concluded that the current methodology is an appropriate tool for advance emergency preparedness and response during a HazMat release in urban environments, and that this is done within practical computer resources.

In a real life situation, the application of source-reconstruction approaches is challenging because of the lack of multiple monitoring stations and of the dependence, amongst others, on the meteorological conditions, emissions, and the released agent characteristics. It is also difficult to collect such information a priori. On the contrary, the proposed methodology is one step closer to the practical applications since it employs symptom-observations as input, instead of concentrations, as in most earlier works. There is room for much future work such as the use of the proposed algorithm with ADREA CFD code for addressing the same problem in urban environments^58,82^. The further investigation of human behaviour during an evacuation from a threatened area to a safe place^83^ and the calculation of toxic load versus the infrastructure effects (e.g. buildings, obstacles, trees, etc) could also be beneficial for the safety engineers.
